# Synergistic Influence of Melatonin-Hydrocolloid Coating on Decay and Senescence of Nectarine (*Prunus persica* var. *nucipersica*) during Supermarket Storage Conditions

**DOI:** 10.3390/plants13060822

**Published:** 2024-03-13

**Authors:** Smruthi Jayarajan, Shruti Sethi, Om Prakash Awasthi, Abhishek Sharma, Danka Bukvički

**Affiliations:** 1 Division of Food Science and Postharvest Technology, ICAR-Indian Agricultural Research Institute, New Delhi 110012, India; docsethi@gmail.com; 2 Amity Institute of Horticulture Studies & Research, Amity University, Noida 201301, India; 3 Division of Fruits and Horticultural Technology, ICAR-Indian Agricultural Research Institute, New Delhi 110012, India; awasthiciah@yahoo.com; 4 Amity Food and Agriculture Foundation, Amity University, Noida 201303, India; 5 Faculty of Biology, Institute of Botany and Botanical Garden ‘Jevremovac’, University of Belgrade, Takovska 43, 11000 Belgrade, Serbia; dankabukvicki@bio.bg.ac.rs

**Keywords:** nectarines, antioxidants, shelf-life, melatonin, hydrocolloid coatings

## Abstract

Nectarines have remarkable nutritional value, low caloric content, and are rich in antioxidants. However, despite substantial local and global demand, their susceptibility to rapid spoilage during peak summer harvest is limited. To address this issue, the current study investigated the potential benefits of using melatonin (MLT), an antioxidant biomolecule, in combination with edible hydrocolloid coatings like carboxymethylcellulose (CMC) and gum Arabic (G.A.) on ‘Snow Queen’ nectarine fruits. The nectarines were treated with various combinations of coatings, including 1% and 1.5% CMC, 8% and 10% G.A., and 0.1 mM melatonin. These coated and non-coated samples were stored under standard supermarket conditions (18 ± 1 °C, 85–90% R.H.) for 16 days. The outcomes demonstrated that the most effective treatment was the combination of 1% CMC with 0.1 mM melatonin. This treatment significantly (*p* ≤ 0.05) reduced the rate of respiration, curbed fruit decay by approximately 95%, minimized weight loss by around 42%, and maintained approximately 39% higher levels of total phenol content and roughly 30% greater antioxidant (AOX) activity. These positive effects were accompanied by preserved firmness and overall quality attributes. Moreover, the treatment extended the shelf life to 16 days through retarding senescence and suppressing the activities of lipoxygenase (LOX) and pectin methylesterase (PME), all without compromising the functional qualities of the nectarine.

## 1. Introduction

Nectarine (*Prunus persica* var. *nucipersica*) is a vital stone fruit belonging to the *Rosaceae* family, cultivated in the lower (warmer) temperate zones around the globe [1]. The commercial cultivation of this crop is still in its infancy in India and is confined to the northeast and northwestern Himalayas. It is a functional fruit containing nearly twice the amount of vitamin A, vitamin B3, potassium, flavonoids, polyphenolic antioxidants like lutein, zeaxanthin, β-cryptoxanthin, and fiber, and is far sweeter than a peach [2,3]. The low calorific value (44 calories/100 g pulp) and the absence of non-saturated fatty acids make this fruit a highly diet-friendly food that any age group can enjoy [4,5].

Based on respiratory and ethylene evolution patterns, nectarine has been categorized as a highly perishable climacteric fruit with a narrow shelf life of about 3–4 days under ambient conditions, yet it can be stored for about 2–3 weeks at cold-storage conditions (0–2 °C, 85–90% R.H.) [4,5]. Ripening is a complex physiological and biochemical process that makes the fruit palatable to the consumers. Polygalacturonase (P.G.) and pectin methylesterase (PME) enzymes are mainly attributed to the softening of fruits [6] and generally show a gradual increase with storage and temperature, finally leading to senescence. Apart from its biochemically enhanced senescence process, the shelf life of the fruit is further reduced drastically due to rough handling, improper transit facility, and poor storage conditions [7]. Furthermore, the fruit’s concise shelf life is also attributed to the shorter time between the commercial ripening and degradative senescence phase [1,4]. The postharvest losses of this fruit, which occur during transportation and market chain, are alarmingly high (25–30%). Thus, special attention is inevitable for postharvest management to prolong nectarine’s shelf life and nutritional quality. 

Several postharvest management strategies are in vogue to improve the postharvest shelf life of fruits through reducing or inhibiting metabolic activities, thereby delaying senescence. The techniques viz. cold storage, modified atmosphere storage [8], use of ethylene absorbents [4,9], use of nitric oxide [6], polyamines, and edible coating [7,10] are commercially practiced worldwide.

Melatonin (N-acetyl-5-methoxytryptamine) (MLT) is a widely studied biomolecule whose functions have been investigated and documented in animals and plants [11,12]. Melatonin has many plant functions, such as promoting seed germination and seedling growth, resistance to various biotic and abiotic stresses, and delays in plant senescence [13,14]. Recent studies have reported that MLT also plays a vital role in reducing fruit decay, delay in ripening and senescence, and improvement in shelf life and quality of stored fruits and vegetables [13,14,15], which depicts the potentiality of MLT as a novel postharvest treatment for preservation of fruits and vegetables. Furthermore, hydrocolloids have long been known to protect perishable food products from deterioration through checking moisture loss, suppressing respiration, improving textural quality, and helping to retain volatile flavor compounds [7,16]. The hydrocolloids are the best edible coatings widely studied/used to control moisture loss and thereby increase the shelf life of fruits [17]. However, a higher decay rate limits its application in highly perishable fruits. Therefore, the study was conducted to evaluate the effect of gum Arabic (G.A.) and carboxymethylcellulose (CMC)-based melatonin-enriched hydrocolloid on the shelf stability of the nectarine stored under supermarket conditions (18 ± 1 °C, 85–90% R.H.).

## 2. Results 

### 2.1. Weight Loss and Percent Decay Incidence

The treatments during storage invariably influenced weight loss (W.L.) and fruit decay in nectarine fruit samples. The combinational treatment of hydrocolloids and melatonin persuaded the weight loss. Regardless of the storage period, T3 (1% CMC + 0.1 mM MLT) reduced W.L. by ~42% over control, which was 5.98% (Table 1). Other treatments also significantly reduced W.L. over control fruits, but the reduction was comparatively less than T3 (Table 1). W.L. increased with the increase in storage period from the fourth to the sixteenth day. The W.L. reported in the samples was highest in control fruits (15.98 ± 0.11%) on the sixteenth day of the storage period and the lowest in T3 (2.74 ± 0.03%) on the fourth day of storage.

**Figure 1:**
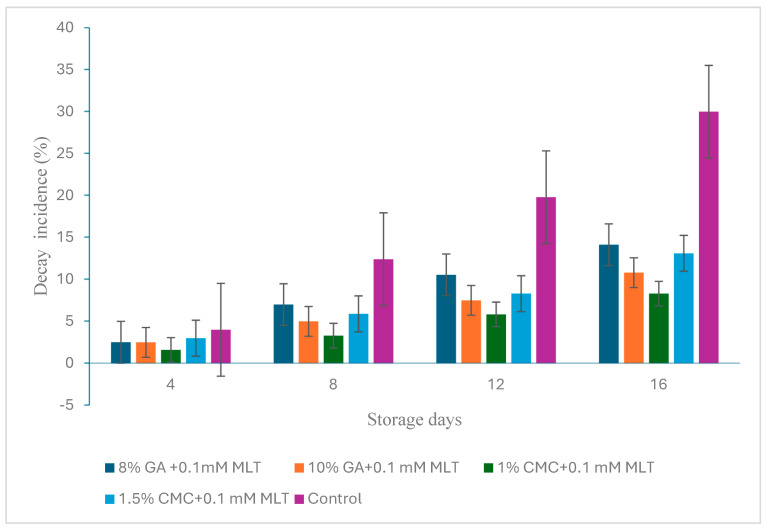
Impact of combined treatments of hydrocolloids and melatonin on decay incidence (%) in ‘Snow Queen’ nectarine fruits during storage at supermarket conditions (18 ± 1 °C and 85–90% R.H.). Data are expressed as mean ± standard errors of triplicate sample at *p* < 0.05.

The combination of hydrocolloids and melatonin had a significant (*p* ≤ 0.05) effect on the decay incidence (%) of nectarine fruits stored at supermarket conditions. The highest fruit decay was recorded in the control samples (16.55%) and the lowest in fruits treated with T3 (4.78%) (Figure 1). These observations revealed that this treatment reduced the fruit decay of nectarine fruits by ~71% over control fruits. The other treatments also significantly reduced fruit decay (*p* ≤ 0.05), but the impact was less significant than T3. The storage period also invariably influenced the decay of fruits, being the highest on the last day (15.27%) and the lowest on the initial day of storage (2.72%). 

### 2.2. Firmness and Respiration Rate

The textural changes in the fruits with the postharvest application of melatonin were studied during the storage and represented in Table 2. The data presented in the table depict that the firmness of the fruits gradually declines with an increase in the storage period, irrespective of treatments. However, the rate of decline differed among treated and controlled fruits. Irrespective of the storage period, maximum firmness was observed in fruits treated with 1% CMC + 0.1 mM MLT (8.23 ± 2.33 N) and minimum in fruits without any treatment (6.88 ± 2.81 N) (Table 2). The storage influenced the firmness of the fruits and there was a gradual progression in the loss of firmness of fruits, regardless of treatments applied. The maximum firmness was recorded on the initial day of storage with a firmness of 10.86 ± 0.34 N and lowest on the last day (16) of storage with 4.06 ± 0.74 N (Table 2). The storage and treatment significantly influenced the texture of the ‘Snow Queen’ nectarine fruit during the storage. The maximum firmness was observed in fruits treated with 1% CMC + 0.1 mM MLT (10.86 ± 0.34 N) during day 0, and the minimum was observed in the last day of storage in fruits stored without any treatment (3.17 N).

**Figure 2:**
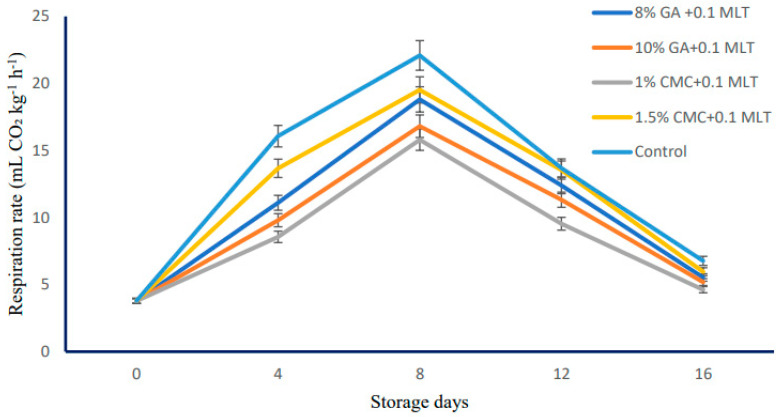
Respiration rate of ‘Snow Queen’ nectarine as influenced by combined treatments of hydrocolloids and melatonin (mL CO_2_ kg^−1^h^−1^) during storage at supermarket conditions (18 ± 1 °C and 85–90% R.H.). Data are expressed as mean ± standard errors of triplicate sample at *p* < 0.05.

The influence of combinational treatment on the respiration rate of ‘Snow Queen’ nectarines during storage is depicted in Figure 2. Being a climacteric fruit, the respiration rate followed a sharp increase, and then it declined further invariably in all treatments and control fruits. The treatments have significantly influenced the respiration rate of the fruits, the lowest being recorded in T3 (1% CMC + 0.1 mM MLT) coated fruits (8.46 mL CO_2_ kg^−1^ h^−1^) and the highest in control fruits (12.45 mL CO_2_ kg^−1^ h^−1^). Regardless of storage, the highest respiratory peak was observed in control fruits (22.09 mL CO_2_ kg^−1^ h^−1^) and the lowest in T3 (15.80 mL CO_2_ kg^−1^ h^−1^) treated fruits. Irrespective of treatments applied, the storage period also influenced the respiratory rate of ‘Snow Queen’ fruits stored in supermarket conditions. The highest respiration rate was observed on the eighth day of storage (18.61 mL CO_2_ kg^−1^ h^−1^) and the lowest on the initial day of storage (3.78 mL CO_2_ kg^−1^ h^−1^). The interaction between storage and treatment has influenced the respiration rate of the fruits significantly. The maximum respiration rate was observed in ‘Snow Queen’ fruits stored without any treatment (22.09 mL CO_2_ kg^−1^ h^−1^) on the eighth day and the lowest in treated as well as control fruits (3.78 mL CO_2_ kg^−1^ h^−1^) on initial days of storage.

### 2.3. Total Phenolic Content (TPC) and Antioxidant Activity

The effect of the postharvest treatment of melatonin-enriched hydrocolloids on the total phenol content of nectarine is presented in Table 3. The total phenolic content of the samples treated with T3 was 39% more than that of the control fruits. Regardless of the treatment, the storage period also influenced the TPC, which was the highest on the fourth day (13.95 ± 1.69 mg GAE 100 g^−1^ FW) and the lowest on the last day (6.74 ± 1.83 mg GAE 100 g^−1^ FW). Other treatments also maintained higher levels of total phenol content but less than those treated with 1% CMC + 0.1 mM MLT. The interaction between the storage period and treatment indicated its significant effect on total phenol content, marking the highest in T3 (15.51 ± 0.54 mg GAE 100 g^−1^ FW) on the fourth day of storage and the lowest in control fruits on the final day of storage (4.16 ± 0.13 mg GAE 100 g^−1^ FW).

The total antioxidant activity of nectarine samples stored under supermarket conditions showed a gradual increase in the initial days of storage and a progressive decline thereafter (Table 4). All the treatments have invariably influenced the total antioxidant activity of nectarine fruits, but T3-treated fruit samples exhibited ~29.7% higher AOX activity over control fruits. The other treatments have also significantly influenced the AOX activity, but their effect was less evident than 1% CMC + 0.1 mM MLT. Regardless of the treatments applied, the storage period significantly influenced the total antioxidant activity of fruit samples during the storage under supermarket conditions, which was the maximum on the fourth day of storage (22.21 ± 2.03 µmol T.E. g^−1^ FW) and the minimum on the last day of storage (11.77 ± 2.16 µmol T.E. g^−1^ FW).

### 2.4. Lipoxygenase and Pectin Methylesterase Activity

The activity of the LOX enzyme showed a rapid increment up to the twelfth day of storage and a non-significant (*p* ≥ 0.05) decline after that (Table 5). The combined treatments of hydrocolloids and melatonin influenced the LOX activity of nectarine fruits during storage. T3-treated fruits exhibited ~34.5% lower LOX activity than control fruits. Fruits coated with other treatments also showed significantly (*p* ≤ 0.05) lower LOX activity, but it was not comparable with treatment. Regardless of treatments, the storage period also had a significant impact on the LOX activity of fruit samples, and it was the minimum on the first day of storage (1.32 ± 0.02 μmol min^−1^ g^−1 ^FW) and the maximum on the twelfth day of storage (3.02 ± 0.54 μmol min^−1^ g^−1 ^FW) (Table 5). The interaction of storage and treatment also significantly impacts LOX activity.

**Figure 3:**
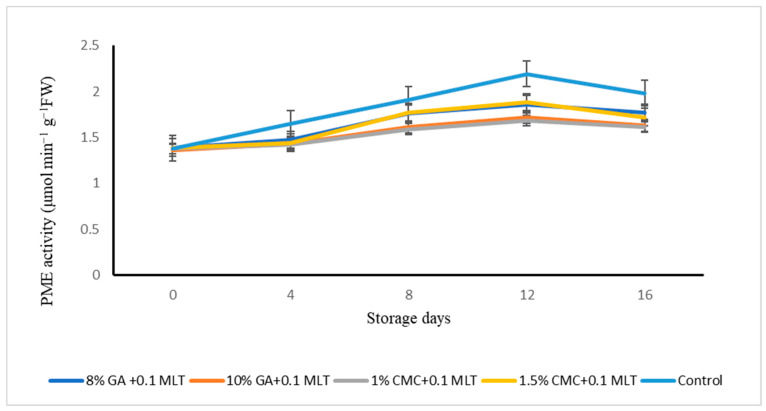
Effect of combined treatments of hydrocolloids and melatonin on pectin methylesterase (PME) activity (µmol min^−1^ g^−1^ FW) of ‘Snow Queen’ nectarine, stored at supermarket conditions (18 ± 1° C and 85–90% R.H.). Data are expressed as mean ± standard errors of triplicate sample at *p* < 0.05.

The activity of the cell-wall-degrading enzyme, pectin methylesterase (PME), showed a significant (*p* ≤ 0.05) increasing trend till the twelfth day of storage, which declined steadily after that (Figure 3) in all treatments. The fruits treated with T3 maintained a lower activity of PME (1.59 ± 0.19 μmol min^−1^ g^−1^ FW) activity than control fruits (1.75 ± 0.26 μmol min^−1^ g^−1^ FW). Other treatments could not exert any significant (*p* ≤ 0.05) influence on the PME activity of nectarine fruits. The storage period also influenced the PME activity of nectarine fruits as the lowest PME activity was recorded on the initial day (0) of storage (1.38 ± 0.01 μmol min^−1^ g^−1^ FW) and the highest on the twelfth day of storage (1.99 ± 0.08 μmol min^−1^ g^−1^ FW). 

### 2.5. Overall Acceptability

The combinational treatment of hydrocolloid and melatonin significantly influenced the overall acceptability score of ‘Snow Queen’ nectarine during storage at supermarket conditions (Table 6). The treatment, storage, and interaction of these factors significantly influence the overall acceptability of fruits. Regardless of the storage period, the fruits treated with T3 maintained a better acceptability score (6.61 ± 0.88) than the control fruits. Furthermore, the storage also influenced the overall acceptability of nectarine fruits, the highest on the eighth day (7.59 ± 0.72) and the lowest at the end of storage (5.75 ± 0.36). The interaction of storage and treatment has influenced the overall acceptability, which was the highest with T3 coated fruits on the eighth day of storage (8.13 ± 0.11) and the lowest on the last day of storage where fruits were without any treatment (4.36 ± 0.05).

## 3. Experimental Materials and Protocol

### 3.1. Procurement of Raw Materials

Research was carried out on nectarines (*Prunus persica* var. *nucipersica*) in 2022 using the fruits of the ‘Snow Queen’ variety. The nectarines were harvested at commercial maturity at the orchard of the Regional Horticultural Research Station, Bajaura Himachal Pradesh, located at 77°7′48″ N latitude and 31°58′56″ longitude. Samples of 40 ripe fruits were collected in each plot out of the three replicates included in each experimental treatment; they were then packed in corrugated fiberboard (CFB) boxes and transported to Delhi under refrigerated conditions for further analysis at the laboratory of the Division of Food Science and Postharvest Technology, ICAR-Indian Agricultural Research Institute, New Delhi. In the lab, the fruits were coated with the following combinations of hydrocolloids and melatonin included in the experimental protocol.

The fruits of the ‘Snow Queen’ variety of nectarine were coated with the combination of hydrocolloid formulation as follows: T1 (8% GA + 0.1 mM melatonin), T2 (10% GA + 0.1 mM melatonin), T3 (1% CMC + 0.1 mM melatonin), T4 (1.5% CMC + 0.1 mM melatonin), and an untreated control; the formulations mentioned above were selected based on our preliminary studies. All these formulations were selected based on a preliminary study conducted using hydrocolloid alone (8% gum Arabic, 10% gum Arabic, 12% gum Arabic, 1.0% CMC, 1.5% CMC [7]) and different concentrations of melatonin alone (0.05 mM, 0.1 mM, 0.5 mM, 1.0 mM). Among the various concentrations applied, 0.1 mM melatonin effectively improved the shelf life of the nectarines concerning biochemical and sensory quality. Hence, 0.1 mM was selected for the combinational treatment. The melatonin was purchased from Sigma Aldrich (St. Louis, MO, USA), and the hydrocolloids were from RANKEM, Avantor Performance Materials, India Limited (Gurgaon, India). The solutions for dipping treatments were prepared in the lab after dissolving the known amount of melatonin in ethanol (98%) and mixing it with the hydrocolloids (aforementioned), prepared with the help of a mechanical stirrer. The fruits dipped in distilled water served as the control. The fruits associated with the experimental treatments selected for T1, T2, T3, and T4 were dipped in their respective treatment solutions at a temperature of 27 °C for 10 min and then air-dried under ambient conditions (32–331 °C) for 3–4 h, whereas the control fruits were dipped in distilled water for 10 min and air-dried. Both the coated samples and control were stored at 18 ± 1 °C and 85–90% R.H. for 16 days. The observations were recorded at an interval of 4 days under supermarket conditions.

### 3.2. Weight Loss

The weight loss was calculated using the following formula and expressed as a percentage (%) [18]. The fruits were weighed regularly (4 days) using an electronic balance.

$$\mathrm{Weight}\;\mathrm{loss}\;\left(\mathrm{WL}\right)\;\left(\%\right)=\frac{\mathrm{Initial}\;\mathrm{weight}-\;\mathrm{Final}\;\mathrm{weight}\;}{\mathrm{Initial}\;\mathrm{weight}}\times 100$$

### 3.3. Firmness

A texture analyzer (model: TA + Di, Stable Microsystems, Godalming, UK) used a compression test [4] to determine the firmness of the’ Snow Queen’ nectarine. The fruit sample was compressed using a probe with programmed speed settings: Pretest: 5 mm/s, Test speed: 0.5 mm/s, and Post-test speed: 10 mm/s. Fruit firmness was expressed in Newtons (N).

### 3.4. Respiration Rate

The respiration rate was determined using an autogas analyzer (Model: Checkmate 9900 O_2_/CO_2_, PBI Dansensor, Ringsted, Denmark) and expressed as mL CO_2_ kg^−1^ h^−1^ [7]. For this, two nectarine fruits were trapped in 1 L airtight containers, and containers were kept at 25 °C for one hour for the accumulation of respiratory gases at the headspace. Using a hypodermic hollow needle, the headspace gas was sucked into the sensor of the analyzer after 1 h, and then the rate of evolution of CO_2_ concentration (%) was recorded. 

### 3.5. Decay Incidence

Fruit decay/loss was calculated by counting the diseased and healthy nectarine fruits in each treatment using the following equation [19].

$$\mathrm{Fruit}\;\mathrm{decay}\;\left(\%\right)=\frac{\mathrm{Number}\;\mathrm{of}\;\mathrm{decayed}\;\mathrm{fruits}\;}{Total\;number\;of\;fruits\;}\;\times \;100$$

### 3.6. Total Phenol Content

The total phenol content of the fruit extracts was determined following the protocols of [20]. Briefly, 2 g of fruit sample was ground in 10 mL of 80% ethanol and then centrifuged at 10,000× *g* for 20 min at 4 °C to obtain the final mixture, and the supernatant was used for further evaluation. In total, 1 mL of the sample was taken in a test tube, and to this, 3 mL of distilled water and 5 mL Folin–Ciocalteau (1 N) was added after 3 min, and 2 mL of 20% Na_2_CO_3_ were also added. Absorbance was measured at 750 nm using a 1 cm cuvette in a Perkin-Elmer UV-VIS Lambda 25 spectrophotometer (Waltham, MA, USA). The total phenol content was expressed in mg of gallic acid equivalents (GAE)/100 g of extract.

### 3.7. Total Antioxidant (AOX) Activity

The antioxidant (AOX) activity in the coated and controlled nectarine was determined using the CUPRAC (Cupric Reducing Antioxidant Capacity) method [21]. For determining antioxidant activity, copper (II) chloride solution, a neoprene alcoholic solution, and an ammonium aqueous buffer (pH 7) were mixed. Then, measurements of the developed color were taken after 30 min in a spectrophotometer at 450 nm. An antioxidant solution for the assay was prepared by accurately weighing 5 g of the sample, which was extracted in 80% ethanol by grinding it in a pestle and mortar and making its volume 15 mL. It was centrifuged at 10,000× *g* for 20 min. The supernatant subsequently collected was used for further analysis. To a test tube, 1 mL each of copper (II) chloride solution (10^−2^ M), neocuprine solution (Nc) of 7.5 × 10^−3^ M, and ammonium acetate (NH_4_Ac) buffer (pH 7) solutions were added. Antioxidant sample (or standard) solution (0.1 mL) and H_2_O (1.0 mL) were added to the initial mixture to make the final volume 4.1 mL. The tubes were then capped, and the absorption at 450 nm was recorded after 30 min. The molar absorptivity of the CUPRAC method for each antioxidant was determined from the slope of the calibration line concerned and was expressed as μmol Trolox (T.E.) g^−1^ fresh weight (FW).

### 3.8. Lipoxygenase (LOX) Activity

The fruit samples were evaluated for the changes in LOX activity over the storage, which was determined using the method of [22]. Briefly, 1 g of fruit pulp was weighed and homogenized in 10 mL of ethylenediamine tetra acetic acid (EDTA) in a pre-chilled pestle and mortar. The homogenate was centrifuged at 15,000× *g* for 20 min at 4 °C, and the supernatant was used to assay lipoxygenase activity. For enzyme assay, 50 µL of enzyme extract was added to 2.975 mL of substrate solution in a cuvette, and readings were recorded for 3 min at 30-s intervals. Absorbance was recorded at 234 nm in a spectrophotometer (Double beam U.V.-VIS spectrophotometer UV5704SS). The blank was prepared by using a substrate solution. LOX activity was expressed as µmoles min^−1^ g^−1^ FW

### 3.9. Pectin Methylesterase (PME) Activity

The pectin methylesterase (PME) activity of the fruit samples was measured following the method of [23], with minor modifications [7]. Briefly, 5 g of fruit pulp was homogenized in 15 mL of 8.8% NaCl maintained at 4 °C, using pestle and mortar. The homogenate was then centrifuged at 15,000× *g* for 15 min. The supernatant was collected, and its pH was adjusted to 7.5 with NaOH, after which it was used for enzyme assay. In a cuvette, 2 mL of pectin was mixed with 0.15 mL of bromothymol blue and 0.83 mL of water. The absorbance of the mixture was read against water as blank at 620 nm. The PME activity was expressed as (0.328 × ∆A_620_ − 0.003) “µmol min^−1^g^−1^ FW”.

### 3.10. Overall Acceptability

The overall acceptability score of the ‘Snow Queen’ nectarine was evaluated with the help of a hedonic scale. The overall acceptability score was based on firmness, color, and appearance. Semi-trained panelists were asked to evaluate the samples on a hedonic scale (0–9) based on overall acceptability in the prescribed intervals [24]. 

### 3.11. Statistical Design and Analysis of Data

The experiments were laid out in a factorial, completely randomized design (CRD), with each treatment with three replicates consisting of 120 fruits with three replications of 40 fruits each. The obtained data related to the measured parameters and were statistically processed mean values for measured parameters that were calculated and compared through analysis of variance using SAS 7.0 software; the mean separations were performed throughout. Storage data values were analyzed using two-way ANOVA followed by a DUNCAN test at *p* < 0.05 [25]. Statistical significance was identified at the 95% confidence level (*p* < 0.05). The average values were reported along with standard deviation.

## 4. Discussion

### 4.1. Weight Loss and Percent Decay Incidence

The data revealed that any combination of hydrocolloids with 0.1 mM melatonin effectively reduced fruit decay without compromising the organoleptic and nutritional quality of fruit samples, but T3 was the most effective. W.L. and respiration rate increased with an increase in storage interval. The treatment T3 maintained ~42% lower W.L. over control fruits during the storage. Physiological/weight loss is a crucial factor in deciding the marketability of fruits in both national and international markets. The stone fruits exceeding 10% of W.L. are unfit for marketing by international standards. The edible coating based on hydrocolloids has been known for its protective action against weight loss for decades [10]. The lower W.L. in combinational treatment of melatonin and hydrocolloid might be attributed to the reduction in respiration rate and transpiration by the physical barrier created by hydrocolloids on drying, with which it regulates the movement of gases and water, effectively maintaining their firmness [7,10]. The higher moisture content of nectarine fruits (85%) makes them highly susceptible to pathogen attack during storage [2]. The fruit samples coated with T3 reduced the decay incidence by more than 95% over control fruits, where it was 8.33%, demonstrating that the treatment worked synergistically for reducing decay invariably in nectarine fruits. This cumulative effect of melatonin, a potent antioxidant, and hydrocolloid might be due to a higher accumulation of the oxidant, H_2_O_2_, which further acts as a trigger for the timely signaling of antioxidant enzyme activity, hence reducing the decay [26,27]. Recent research has shown the positive interaction between melatonin and signaling molecules; for instance, the exogenous melatonin induced the biotic stress response in nectarine to combat stress [12]. In addition, the phenylpropanoid pathway might have provided a better defense mechanism in melatonin and hydrocolloid-coated fruits, which reduced fruit decay [26].

Hydrocolloids also act as a physical barrier between host and pathogen; hence, they might also have acted synergistically with melatonin to reduce the decay incidence [10,17]. The melatonin-treated (1000 µM) plums stored at cold storage showed a more significant reduction in decay incidence than control plums [28]. Similarly, Botrytis rot in tomatoes was controlled by exogenous melatonin application, which activated the jasmonic acid signaling pathway, thereby inducing higher resistance in the treated fruits [29].

### 4.2. Firmness and Respiration Rate

The firmness of the fruits showed a declining trend with the advancement of the storage period, and the minimum firmness was recorded in fruits stored without coating and the maximum in fruits with T3 coating. The decline in firmness is mainly attributed to the softening of the cell wall by pectin methyl esterase and polygalacturonase, which increases with the advancement of storage. Firmness is the most crucial factor that decides the acceptance of fruit in the market and is associated with changes in cell wall structure caused by various cell-wall-degrading enzymes. The better firmness of the coated fruits might be due to the protective physical barrier created by hydrocolloid coatings such as G.A. and CMC, creating an uncongenial environment that resulted in reduced respiration and transpiration and delays in the softening of the cell wall. Our study aligns with the findings of [30], whose results showed that citrus fruits coated with CMC have extended shelf life with lower weight loss and better firmness than control fruits. Also, ref. [24] reported that apple fruits treated with aloe vera and CMC have better physio-chemical properties than control fruits.

The respiration rate of the ‘Snow Queen’ nectarine increased during the first few days (eighth day) and then declined afterward, which depicted the climacteric nature of the nectarine fruit. The respiration rate of the fruit was significantly influenced by the treatment during storage (Table 6). The highest was observed in control fruits and the lowest in fruits coated with T3. The lower respiration rate in coated fruits might be due to the coating, which creates higher CO_2_ around the fruit, leading to a reduced respiration rate [31]. Our study supports Prasad et al. [17], who revealed that mangoes coated with 1% CMC exhibited lower respiration rates, less weight loss, and reduced darkening. Similarly, Jhalegar et al. [31] reported that coated ‘Kinnow’ mandarin fruits exhibited the lowest physiological loss in weight, fruit firmness, respiration rate, and ethylene evolution rate. Melatonin has also shown efficacy in controlling the respiration rate in pears, mangoes, and bananas by regulating TCA cycle-related genes [32].

### 4.3. Total Phenolic Content (TPC) and Antioxidant Activity

Table 2 and Table 3 data revealed that the combinational treatment of melatonin and hydrocolloids significantly (*p* ≤ 0.05) influenced the functional attributes such as the total phenol content and antioxidant activity of nectarine fruits. The treated fruits, especially with T3, maintained a total phenol content 39% higher and a total antioxidant activity 30% higher than control fruits (Table 2 and Table 3). The higher biochemical/functional attributes in melatonin-treated fruits might be due to the upregulation of various genes involved in producing these biochemicals, mainly conferred as the first line of defense [26,27]. Furthermore, the higher retention of total phenols might be attributed to the lower oxidation of phenolic components in coated fruits by the higher melatonin content [27]. Applying hydrocolloids on the fruit surface creates a modified atmosphere around the fruit, resulting in lower respiration and oxidation of phenols through suppressing the enzymes involved, such as PPO [32]. Gol et al. [33] also reported that the application of chitosan (C.H.), gum Arabic (G.A.), and alginate (A.L.) affected the shelf life and overall quality of carambola fruits by maintaining higher concentrations of total phenolics and antioxidant activity.

### 4.4. Lipoxygenase and Pectin Methylesterase Activity

Enzymes such as PME and LOX play a vital role in fruit’s senescence and postharvest life. The cell wall-degrading enzymes like PME and P.G. become activated with higher carbon dioxide as they progress through ripening, leading to senescence. On the other hand, LOX leads to the peroxidation of PUFA, leading to a loss in compartmentation and cell breakdown [33,34]. The combination treatment of melatonin and hydrocolloids (1% CMC + 0.1 mM melatonin) lowered the activities of the cell-wall-degrading enzyme, PME (Figure 2), and LOX significantly (Table 4) in nectarine fruits. The lower activity of PME and LOX in 1% CMC + 0.1 mM melatonin (T3) treated nectarine fruits might be attributed to lower respiration rate, a metabolic activity responsible for delaying fruit senescence [15]. Hydrocolloid-based coatings [7,10] and melatonin [15,23] have been reported to inhibit the ripening and senescence in fruits, and hence their combination in this study worked synergistically for reducing the senescence process of nectarine fruits. The exogenous melatonin application downregulated the expression of cell-wall-degrading enzymes such as PME and LOX, contributing to better cell wall integrity [35]. Liu et al. [34] elucidated the role of exogenous melatonin on the ripening and senescence of mango by controlling the activities of the cell-wall-softening enzymes like PME and LOX.

### 4.5. Overall Acceptability

The overall acceptability of ‘Snow Queen’ fruits coated with T3 was superior throughout the storage. Prasad et al. [17] reported that fruits coated with hydrocolloids showed better sensory quality due to higher TSS maintenance, better firmness, and overall eating quality.

## 5. Conclusions

The combined treatment of melatonin and hydrocolloids significantly reduced nectarine fruits’ weight loss and decay incidence during storage at supermarket storage conditions. Dipping nectarine fruits with 1% CMC + 0.1 mM melatonin invariably increased shelf life to 16 days by maintaining acceptable weight loss (W.L.), better firmness, and other biochemical attributes such as antioxidants and phenols. The decay, which was the major drawback of hydrocolloid treatment, was successfully alleviated by the incorporation of potential antioxidant melatonin into the coating matrix. The senescence was deferred in all the treatments effectively by retaining the higher functional attributes like phenolics, antioxidants, and antioxidant enzymes and lowering the activities of cell wall degrading and senescence hastening enzymes such as PME and LOX. The outcome arisen from this research may be useful for commercial application to increase the storage life of nectarine fruits under supermarket conditions. 

## Figures and Tables

**Table 1:** Weight loss (%) of nectarine fruits * as influenced by combined treatments of hydrocolloids and melatonin during storage at supermarket conditions (18 ± 1 °C and 85–90% R.H.).

Treatment	Storage Period (Days)				Mean
	4	8	12	16	
8% G.A. + 0.1 mM MLT	3.40 ± 0.2 ^bD^	6.80 ± 0.10 ^bC^	9.50 ± 0.10 ^bB^	12.84 ± 0.20 ^bA^	8.13 ± 3.62
10% GA + 0.1 mM MLT	2.74 ± 0.03 ^cD^	5.10 ± 0.10 ^dC^	7.80 ± 0.20 ^dB^	10.06 ± 0.02 ^dA^	6.41 ± 2.86
1% CMC + 0.1 mM MLT	2.81 ± 0.02 ^cD^	4.23 ± 1.33 ^eC^	7.19 ± 0.01 ^eB^	9.71 ± 0.05 ^eA^	5.98 ± 2.84
1.5% CMC + 0.1 mM MLT	3.44 ± 0.01 ^bD^	6.00 ± 0.20 ^cC^	9.03 ± 0.06 ^cB^	12.42 ± 0.05 ^cA^	7.72 ± 3.50
Control	4.56 ± 0.35 ^aD^	8.10 ± 0.20 ^aC^	12.92 ± 0.10 ^aB^	15.98 ± 0.11 ^aA^	10.39 ± 4.58
Mean	3.39 ± 0.69	6.04 ± 1.47	9.28 ± 2.06	12.19 ± 2.34	

* In each treatment there are 120 fruits with three replications of 40 fruit each. In each row data followed by same small letters are not significantly different at *p* < 0.05. In each column data followed by same capital letters are not significantly different at *p* < 0.05.

**Table 2:** Impact of combination treatment of hydrocolloids and melatonin on firmness (N) of ‘Snow Queen’ nectarine fruits * during storage at supermarket conditions (18 ± 1 °C and 85–90% R.H.).

Treatment	Storage Period (Days)					Mean
	0	4	8	12	16	
8% G.A. + 0.1 mM MLT	10.76 ± 0.34 ^aA^	8.48 ± 0.34 ^bB^	6.91 ± 0.26 ^bC^	5.22 ± 0.20 ^cC^	3.63 ± 0.30 ^dC^	7.00 ± 2.58
10% GA + 0.1 mM MLT	10.61 ± 0.34 ^aA^	9.75 ± 0.36 ^aA^	7.67 ± 0.18 ^bB^	6.48 ± 0.18 ^bB^	4.50 ± 0.29 ^cB^	7.80 ± 1.98
1% CMC + 0.1 mM MLT	10.86 ± 0.34 ^aA^	9.45 ± 0.39 ^aA^	8.41 ± 0.20 ^bA^	7.29 ± 0.18 ^bA^	5.17 ± 0.11 ^cA^	8.23 ± 2.33
1.5% CMC + 0.1 mM MLT	10.16 ± 0.34 ^aA^	9.49 ± 0.30 ^aA^	6.97 ± 0.09 ^bC^	5.78 ± 0.30 ^bC^	3.83 ± 0.07 ^cC^	7.24 ± 2.59
Control	10.46 ± 0.34 ^aA^	9.02 ± 0.13 ^aB^	6.76 ± 0.10 ^bC^	5.03 ± 0.06 ^cD^	3.17 ± 0.18 ^dD^	6.88 ± 2.81
Mean	10.57 ± 0.29	9.23 ± 0.53	7.34 ± 0.65	5.96 ± 0.88	4.06 ± 0.74	

* In each treatment there are 120 fruits with three replications of 40 fruit each. In each row data followed by same small letters are not significantly different at *p* < 0.05. In each column data followed by same capital letters are not significantly different at *p* < 0.05.

**Table 3:** Influence of combined treatment of hydrocolloids and melatonin on total phenol content (mg GAE 100 g^−1^ FW) of ‘Snow Queen’ nectarine fruits * during storage at supermarket conditions (18 ± 1 °C and 85–90% R.H.).

Treatment	Storage Period (Days)					Mean
	0	4	8	12	16	
8% G.A. + 0.1 mM MLT	13.00 ± 0.03 ^cB^	14.59 ± 0.02 ^cA^	12.19 ± 0.08 ^bC^	9.52 ± 0.15 ^cD^	7.75 ± 0.07 ^cE^	11.41 ± 2.54
10% GA + 0.1 mM MLT	13.47 ± 0.03 ^aB^	14.80 ± 0.05 ^bA^	11.80 ± 0.04 ^cC^	9.15 ± 0.34 ^bD^	7.88 ± 0.04 ^bE^	11.42 ± 2.60
1% CMC + 0.1 mM MLT	13.32 ± 0.03 ^bC^	15.51 ± 0.54 ^aA^	14.15 ± 0.11 ^aB^	10.71 ± 0.0 ^aD^	8.78 ± 0.31 ^aE^	12.51 ± 2.49
1.5% CMC + 0.1 mM MLT	13.43 ± 0.03 ^aB^	13.79 ± 0.10 ^dA^	10.16 ± 0.09 ^dC^	8.20 ± 0.14 ^dE^	5.16 ± 0.13 ^dF^	10.15 ± 3.04
Control	13.41 ± 0.03 ^aA^	11.04 ± 0.03 ^eB^	9.92 ± 0.08 ^eC^	6.50 ± 0.28 ^eD^	4.16 ± 0.13 ^eE^	9.01 ± 3.29
Mean	13.32 ± 0.02	13.95 ± 1.69	11.64 ± 1.58	8.83 ± 1.49	6.74 ± 1.83	

* In each treatment there are 120 fruits with three replications of 40 fruit each. In each row data followed by same small letters are not significantly different at *p* < 0.05. In each column data followed by same capital letters are not significantly different at *p* < 0.05.

**Table 4:** Synergistic effect of hydrocolloids and melatonin on total antioxidant activity (µmol T.E. g^−1^ FW) of ‘Snow Queen’ nectarine fruits * stored at supermarket conditions (18 ± 1 °C and 85–90% R.H.).

Treatment	Storage Period (Days)					Mean
	0	4	8	12	16	
8% GA + 0.1 mM MLT	20.12 ± 0.09 ^aC^	23.04 ± 0.18 ^bA^	20.99 ± 0.06 ^bB^	17.41 ± 0.54 ^bD^	12.60 ± 0.04 ^bE^	18.83 ± 3.73
10% GA + 0.1 mM MLT	20.12 ± 0.09 ^aC^	22.77 ± 0.09 ^cA^	20.78 ± 0.10 ^cB^	17.47 ± 0.05 ^bD^	12.41 ± 0.06 ^eE^	18.71 ± 3.70
1% CMC + 0.1 mM MLT	20.12 ± 0.09 ^aC^	24.92 ± 0.95 ^aA^	23.22 ± 0.11 ^aB^	19.15 ± 0.10 ^aD^	14.76 ± 0.12 ^aE^	20.43 ± 3.65
1.5% CMC + 0.1 mM MLT	20.12 ± 0.09 ^aB^	21.18 ± 0.10 ^dA^	18.27 ± 0.04 ^dC^	14.51 ± 0.20 ^cD^	10.40 ± 0.10 ^dE^	16.89 ± 4.10
Control	20.12 ± 0.09 ^aA^	19.15 ± 0.05 ^eB^	17.29 ± 0.06 ^eC^	13.54 ± 0.12 ^dD^	8.67 ± 0.07 ^eE^	15.75 ± 4.34
Mean	20.12 ± 0.07	22.21 ± 2.03	20.11 ± 2.18	16.42 ± 2.15	11.77 ± 2.16	

* In each treatment there are 120 fruits with three replications of 40 fruit each. In each row data followed by same small letters are not significantly different at *p* < 0.05. In each column data followed by same capital letters are not significantly different at *p* < 0.05.

**Table 5:** Activities of lipoxygenase (μmol min^−1^ g^−1^ FW) as influenced by combined treatment of hydrocolloids and melatonin in ‘Snow Queen’ nectarine fruits * stored in supermarket conditions (18 ± 1 °C and 85–90% R.H.).

Treatment	Storage Period (Days)					Mean
	0	4	8	12	16	
8% G.A. + 0.1 mM MLT	1.33 ± 0.02 ^aE^	1.77 ± 0.01 ^cD^	2.42 ± 0.25 ^cC^	3.08 ± 0.11 ^cA^	2.78 ± 0.12 ^cB^	2.28 ± 0.67
10% GA + 0.1 mM MLT	1.31 ± 0.01 ^aE^	1.72 ± 0.02 ^dD^	2.36 ± 0.01 ^dC^	2.72 ± 0.03 ^dA^	2.42 ± 0.02 ^dB^	2.11 ± 0.52
1% CMC + 0.1 mM MLT	1.32 ± 0.02 ^aE^	1.64 ± 0.06 ^eD^	2.06 ± 0.06 ^eB^	2.24 ± 0.03 ^eA^	1.94 ± 0.05 ^eC^	1.84 ± 0.33
1.5% CMC + 0.1 mM MLT	1.34 ± 0.02 ^aE^	1.93 ± 0.03 ^bD^	2.76 ± 0.04 ^bC^	3.28 ± 0.05 ^bA^	2.98 ± 0.05 ^bB^	2.45 ± 0.74
Control	1.33 ± 0.02 ^aE^	2.42 ± 0.11 ^aD^	3.06 ± 0.10 ^aC^	3.79 ± 0.02 ^aA^	3.49 ± 0.02 ^Ab^	2.81 ± 0.90
Mean	1.32 ± 0.02	1.89 ± 0.2	2.53 ± 0.37	3.02 ± 0.54	2.72 ± 0.54	

* In each treatment there are 120 fruits with three replications of 40 fruit each. In each row data followed by same small letters are not significantly different at *p* < 0.05. In each column data followed by same capital letters are not significantly different at *p* < 0.05.

**Table 6:** Influence of combination treatment of hydrocolloids and melatonin on overall acceptability score of ‘Snow Queen’ nectarine fruits * during storage in supermarket conditions (18 ± 1° C and 85–90% R.H.).

Treatment	Storage Period (Days)					Mean
	0	4	8	12	16	
8% G.A. + 0.1 mM MLT	5.54 ± 0.01 ^aC^	5.74 ± 0.02 ^Eb^	6.25 ± 0.01 ^eA^	5.40 ± 0.20 ^dD^	5.30 ± 0.10 ^eD^	5.64 ± 0.35
10% GA + 0.1 mM MLT	5.53 ± 0.01 ^aD^	6.50 ± 0.10 ^bB^	8.00 ± 0.20 ^bA^	6.10 ± 0.10 ^bC^	6.11 ± 0.01 ^bC^	6.45 ± 0.86
1% CMC + 0.1 mM MLT	5.56 ± 0.03 ^aD^	6.77 ± 0.15 ^aB^	8.13 ± 0.11 ^aA^	6.30 ± 0.10 ^aC^	6.30 ± 0.20 ^aC^	6.61 ± 0.88
1.5% CMC + 0.1 mM MLT	5.54 ± 0.01 ^aD^	6.63 ± 0.15 ^cB^	7.70 ± 0.20 ^cA^	5.83 ± 0.05 ^cC^	5.83 ± 0.15 ^cC^	6.30 ± 0.81
Control	5.47 ± 0.01 ^aE^	6.40 ± 0.10 ^dB^	7.10 ± 0.20 ^dA^	5.21 ± 0.20 ^eC^	4.36 ± 0.05 ^eD^	5.50 ± 0.84
Mean	5.55 ± 0.01	6.40 ± 0.38	7.59 ± 0.72	5.92 ± 0.34	5.75 ± 0.36	

* In each treatment there are 120 fruits with three replications of 40 fruit each. In each row data followed by same small letters are not significantly different at *p* < 0.05. In each column data followed by same capital letters are not significantly different at *p* < 0.05.

## Data Availability

Data are contained within the article.
